# Detection of urban trees sensitivity to air pollution using physiological and biochemical leaf traits in Tehran, Iran

**DOI:** 10.1038/s41598-022-19865-3

**Published:** 2022-09-13

**Authors:** Hamed Dadkhah-Aghdash, Milad Rasouli, Kabir Rasouli, Azam Salimi

**Affiliations:** 1 Department of Plant Biology, Faculty of Biological Sciences, Tarbiat Modares University, Tehran, Iran; 2 Endocrinology and Metabolism Research Center, Endocrinology and Metabolism Clinical Sciences Institute, Tehran University of Medical Sciences, Tehran, Iran; 3 Department of Physics, Kharazmi University, Tehran, Iran; 4 Department of Geography, The University of British Columbia, Vancouver, BC Canada; 5 Department of Plant Science, Faculty of Biological Sciences, Kharazmi University, Tehran, Iran

**Keywords:** Plant sciences, Environmental sciences, Environmental impact

## Abstract

The increased population in megacities has recently exacerbated the need to combat air pollution. This study examined the concept that the sensitivity and tolerance of urban plant species to air pollution might be used to determine Tehran, Iran's air quality and obtain suitable urban greening. The air pollution tolerance index (APTI) was derived using the total chlorophyll, relative water content, pH, and ascorbic acid content of leaf extract from *Morus alba*, *Ailanthus altissima*, and *Salix babylonica* trees as an indicator of the sensitivity and tolerance of urban plant species. *A. altissima* and *S. babylonica*, with APTI values of 11.15 and 11.08, respectively, were sensitive to air pollution and can be employed as bioindicators, whereas *M. alba*, with an APTI value of 14.08, exhibited moderate resistance to air pollution and is therefore recommended for urban planting. Furthermore, the content of enzymatic and non-enzymatic parameters (carotenoid, phenol, and flavonoids) and proline concentration in the polluted seasons and sites (3 and 4) have been increased in *M. alba*. Collectively, we expect our findings to contribute to the rapidly growing body of research aiming to find a suitable urban greening for a wide range of polluted megacities.

## Introduction

Air pollution is the main cause of various diseases, and it is one of the most concerning environmental issues, causing more than 40,000 deaths each year^1,2^. Among diverse air pollutants, particulate matter (PM), nitric oxide (NO), lead (Pb), and carbon monoxide (CO) are the most dangerous pollutants, which have negative impacts on humans, animals, and plant species^3^. By filtering air pollution and reducing human exposure to anthropogenic contaminants, tree planting in urban settings is a low-cost, effective strategy^4^. Tree parts such as leaves and bark can absorb air pollutants, and they are good proxies to assess the status of air quality in polluted urban areas and parks^3^.

One of the parameters related to the responses of plant species is the air pollution tolerance index (APTI). It consists of total chlorophyll, relative water content, pH of leaf extracts, and ascorbic acid, and it is used to indicate plants sensitivity or tolerance to air pollution^5^. When plants are exposed to air pollutants, reactive oxygen species (ROS) are enhanced, which first leads to oxidative stress and eventually causes the cell death procedure^6,7^. Different mechanisms are used for detoxification of ROS in plants, including a) activating enzymatic antioxidants such as catalase, ascorbate peroxidase (APX), and phenylalanine ammonia-lyase (PAL); b) activating hydrophilic (such as ascorbate and glutathione) and lipophilic (carotenoid) compounds^8–10^. According to Krishnaveni^11^, phenol and flavonoid compounds are utilized as antioxidants as their amounts change under oxidative stresses and the scavenging of ROS. When plants are subjected to abiotic stress, proline is accumulated in their tissues with osmoprotectant activities^12^. Proline has four functions: (a) alimental storage for growth, (b) proteins and membrane stabilization, (c) the scavenging of free radicals, and (d) acting as an antioxidant system^13–15^.

According to Alotaibi et al.^16^, the responses of five tree species to air pollution were assessed in Riyadh City, Saudi Arabia. The APTI value of the trees in four sites was measured and the highest tolerance of them, which is *Ficus altissima* Benth, was proposed to be applied in the green spaces of the city. Manjunath and Reddy^17^ reported that *Bougainvillea spectabilis* willd. and *Vinca rosea* L. plants had the highest tolerance to the pollution stressors and can be used in urban areas to develop greenbelts. In two urban regions of Tehran, the physiological responses, including proline, protein content, and activity of the nitrate reductase enzyme, of *Laurus nobilis* L. to air pollution were studied^18^. Additionally, in response to the urban air pollution by heavy metals (Cd, Pb, Ni, Zn, and Cr), the anatomical and certain physiological features (pigment contents and enzymatic antioxidants) of *Platanus orientalis* L. leaves in two urban areas of Tehran were measured^19^.

Tehran is one of the most air-polluted and most populated cities in western Asia. Urbanization, industrial development, consumption of low-quality fuels, rapid population growth, and the topography of the city are the main causes of air pollution in Tehran. Particulate matter (PM_2.5_) is the most significant air pollutant, with an annual concentration three times higher than the maximum range of national and WHO standards. PM poses high health risks by penetrating the lungs and entering the bloodstream^20^. Therefore, creating appropriate green areas and parks through choosing tolerant trees, which can control air pollution in Tehran, is vital.

This study aims to examine the reactions of trees to air pollution in several Tehran locations during the spring, summer, and autumn to obtain a suitable urban greening for controlling air pollution in this city. To determine tolerant and sensitive trees, the effects of air pollution on the leaves of *Morus alba*, *Ailanthus altissima*, and *Salix babylonica*, which are common deciduous trees in Tehran, were studied. These three tree species are utilized in the urban ecosystems of Iran due to their high absorption capacity for air pollutants and their ability to avoid various types of environmental pollution, as well as their economic and social benefits for the city's inhabitants^4,21^. The hypothesis is that trees with high APTI values are more resistant to air pollution and more suitable for urban greening than trees with low APTI values. The question is what the link is between the APTI value, the activity and content of antioxidants (enzymatic and non-enzymatic) and the proline concentration of various tree species in various regions and seasons. To identify tolerant and sensitive trees, the effects of air pollution on the leaves of *Morus alba*, *Ailanthus altissima*, and *Salix babylonica*, three common deciduous trees in cities, were investigated using antioxidant activity and content (enzymatic and non-enzymatic), as well as proline concentration. The APTI index was calculated using the overall chlorophyll content, relative water content, ascorbic acid concentration, and pH. Based on the APTI values, trees are classified as sensitive or tolerant. Based on the APTI values, trees are classified as sensitive or tolerant and recommended for future urban greening of megacities.

## Material and methods

### Study area

**Figure 1:**
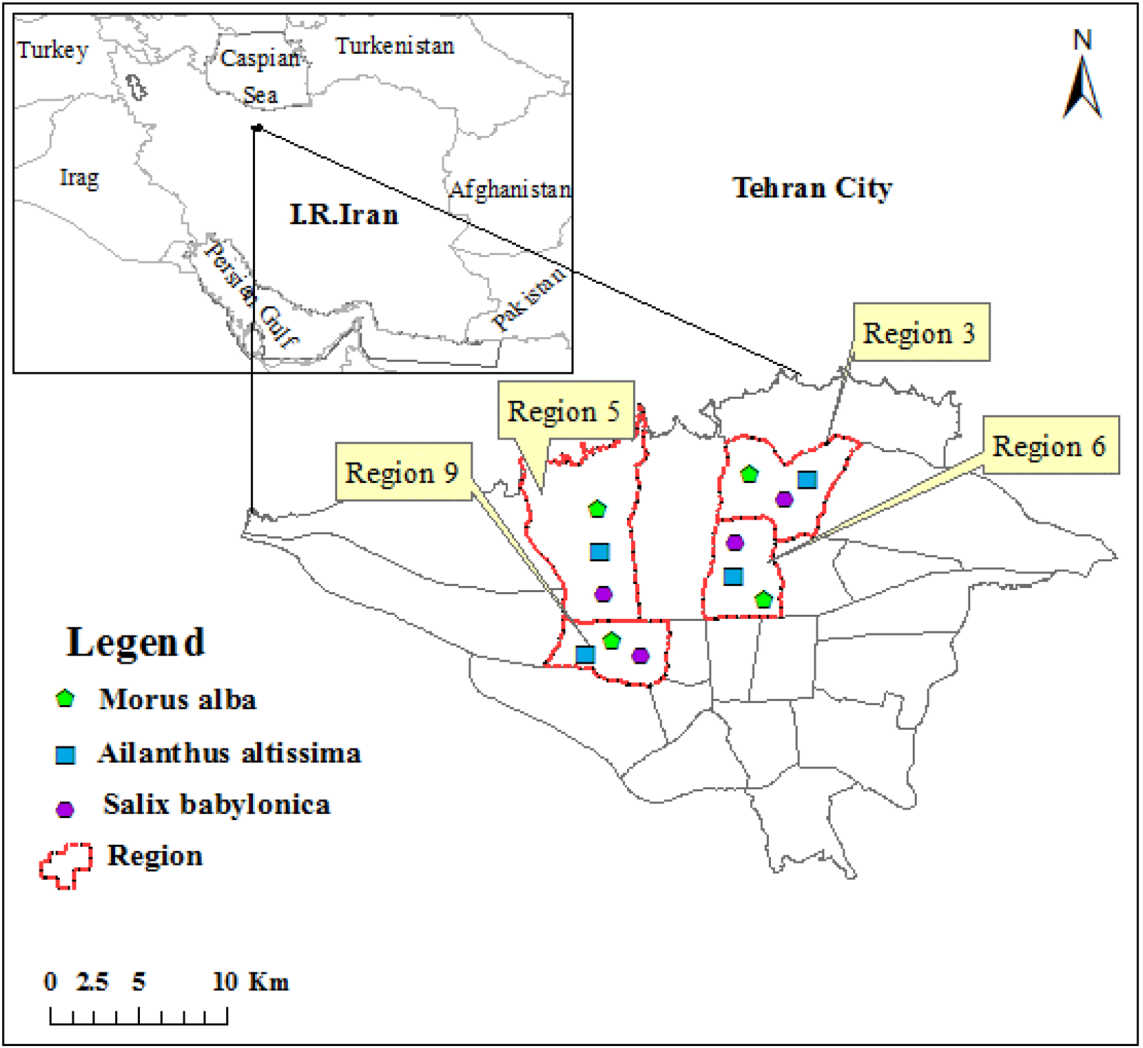
Map of the study sites in Tehran, Iran (drawn by H. Dadkhah-Aghdash using software of ArcGIS Desktop. version of 10.5. ESRI, California, US. https://desktop.arcgis.com).

Tehran is one of the largest Middle Eastern megacity^22,23^, which has 22 regions with different air pollution monitoring stations in each region. The air quality index on the different days of plant species sampling has shown in the supplementary file. Given the information from air pollution monitoring stations data in the city, four sites were selected for sampling tree species in the different seasons of the year, including Mirdamad Street (region 3 or site 1), Kargar Shomali Street (region 6 or site 3), and Azadi Bus Terminal Street (region 9 or site 4) as polluted sites, and Mirza Babaei Street (region 5 or site 2) as a control site. The map of study area was drawn by software of ArcGIS version of 10.5, https://desktop.arcgis.com (Fig. 1). Mirza Babaei Street and Azadi Bus Terminal had the lowest and highest levels of air pollution, respectively, among the studied districts.

### Plant samples collection

All procedures were followed in compliance with institutional, national, and international rules and legislation. The formal identification of *Morus alba* L. (white mulberry), *Ailanthus altissima* (Mill.) Swingle (tree-of-heaven), and *Salix babylonica* L. (willow) was performed based on the colorful flora of Iran^24^. Permissions or licenses were secured to collect these trees in the Tehran metropolis. These trees voucher specimens were gathered and deposited in a herbarium with the Department of Plant Biology, Tarbiat Modares University.

A total of 270 samples (three different tree species × three trees of each species × ten leaves from each tree × three seasons of the year) were sampled at each studied site. It is noteworthy that leaf samples were collected from different levels and heights of canopy cover trees with equal age and breast diameter in late spring (May 31), summer (September 22), and autumn (November 15) in 2019. Further details of the trees can be found in Supplementary Information (Figure S1). Details of the concentration of air pollutants on sampling days can be found in Supplementary Information (Figures S2, S3, and S4). Leaf samples were transported to the laboratory using a portable ice box for biochemical and physiological analysis. Leaf samples were washed and analyzed on the same day and were stored at −80 °C in the freezer^5^.

This study aims to examine the reactions of trees to air pollution in several Tehran locations during the spring, summer, and autumn. During these seasons, leaf samples from the examined trees were gathered. The total chlorophyll content, relative water content, ascorbic acid concentration, and pH were determined. The APTI value was computed using these four parameters. According to the obtained APTI value, the studied trees in the summer and autumn had a greater degree of tolerance than in the spring. On the other hand, according to the air quality index established by air pollution monitoring stations of the city, the spring season had relatively good and clean air quality compared to the summer and autumn seasons. Due to the aforementioned factors, the biochemical parameters associated with air pollution stress tolerance, including enzymatic and non-enzymatic antioxidants and proline, were examined in the summer and autumn seasons.

## The air pollution tolerance index assays

### Total chlorophyll content assay

Leaf samples with similar shapes and ages were selected for biochemical and physiological analyses. Crushed fresh leaves were extracted in 80% (v/v) acetone, and light absorbance at 646.8 and 663.2 nm was measured using a spectrophotometer (Perkin Elmer-USA). Acetone 80% was used as a control for the solution. Calculations were conducted using the following equation^25^:

1 $$\begin{gathered} {\text{Chl}}.{\text{a }}\left(\upmu {{\text{g ml}}^{{ - {1}}} {\text{solution}}} \right) \, = {12}.{\text{25 A}}_{{{663}.{2}}} - { 2}.{\text{79 A}}_{{{646}.{8}}} \hfill \\ {\text{Chl}}.{\text{b }}(\upmu {\text{g ml}}^{{ - {1}}} {\text{solution}}) \, = {21}.{5}0{\text{ A}}_{{{646}.{8}}} - { 5}.{1}0{\text{ A}}_{{{663}.{2}}} \hfill \\ {\text{Chl}}.{\text{T }} = \, \left( {{\text{Chl}}.{\text{a }} + {\text{ Chl}}.{\text{b}}} \right) \hfill \\ \end{gathered}$$

where A_663.2_ and A_646.8_ refer to the absorbance values of the corresponding wavelengths. Chl T is the total chlorophyll concentration of the solution. Chlorophyll content was expressed according to the volume of the extract and the weight of the samples based on milligrams per gram of fresh weight (mg g^−1^ FW).

### Relative water content (RWC) assay

2 $${\text{RWC }} = \, \left[ {\left( {{\text{FW}} - {\text{ DW}}} \right)/ \, \left( {{\text{TW }}{-}{\text{DW}}} \right)} \right] \times {1}00$$

where RWC is relative water content (%), was calculated according to the equation of 2. FW is the fresh weight (g), DW is the dry weight (g) of leaves under 3 h oven-drying treatment at 105 °C and TW is the leaf turgid weight (g) obtained by overnight immersion in deionized water^26,27^.

### pH of leaf extracts assay

To measure the pH of the leaf extract, 0.5 g of fresh leaves were crushed and homogenized in 50 ml of deionized water and centrifuged at 3000 rpm for 15 min. The supernatant pH was determined using a digital pH meter (Fan Azma Gostar Company, Iran).

### Ascorbic acid concentration assay

The ascorbic acid concentration was measured according to the method reported by Kampfenkel et al.^28^. Fresh leaves weighing as much as 0.5 g were grinded with 10 ml of 5% metaphosphoric acid solution, and the mixture was centrifuged at 10,000*g* for 15 min. Then, 0.3 ml of a centrifuged extract was thoroughly mixed with 0.75 ml of potassium phosphate buffer and 0.3 ml of distilled water by Vortex for 10 min at room temperature. The solution was incubated for 40 min at 40 °C with 10% Trichloroacetic acid (TCA), 0.6 ml of 44% Orthophosphoric acid, 0.6 ml of 4% Dipyridyl, and 10 L of FeCl_3_. Finally, the light absorbance was read at 525 nm by a spectrophotometer and the ascorbic acid content was calculated using the standard curve in mg g^−1^ fresh weight.

### Air pollution tolerance index determination

APTI of plant species is determined by the formula introduced by^29,30^ as following:

3 $${\text{APTI }} = \, \left( {\left[ {{\text{A }}\left( {{\text{T }} + {\text{ P}}} \right)} \right] \, + {\text{ R}}} \right) \, /{ 1}0$$

A is the ascorbic acid content of the leaf in mg g^−1^ FW; T is the total chlorophyll content in mg g^−1^ FW, P is the pH of the leaf extract, and R is the percentage of relative water content. Plants with APTI values ranging between 17–30, 12–16, 1–11, and < 1 were considered tolerant, intermediate tolerant, sensitive, and very sensitive, respectively^31^.

### The assay of enzymatic antioxidants

Fresh leaf samples (0.5 g) were homogenized in 0.8 mL of 100 mM potassium-phosphate buffer (pH 7) and then were centrifuged at 15,000*g* for 15 min. The supernatants were used for the determination of enzyme activities.

### Assessment of catalase activity

According to the method of Dhindsa et al.^32^, the decrease of hydrogen peroxide absorbance at 240 nm was measured for 1 min. The reaction mixture is 3 mL (containing 2800 μL of 50 mM potassium phosphate buffer (pH 7.0), 100 μL of 15 mM hydrogen peroxide, and 100 μL of the enzyme extract). The reduction in the amount of hydrogen peroxide per minute at a wavelength of 240 nm using a spectrophotometer is considered the unit of enzymatic activity.

### Assessment of ascorbate peroxidase activity

The reaction mixture contains 50 mM potassium phosphate buffer (pH 7), 0.5 mM ascorbate, 0.1 mM hydrogen peroxide, and 150 mM enzyme extract, which was measured with a spectrophotometer at 290 nm for 1 min based on ascorbic acid oxidation and decrease^33^.

### Assessment of phenylalanine ammonia-lyase activity

According to the method of D'Cunha et al.^34^, about 600 μL of 50 mM Tris buffer (pH 8.8) was used with 900 μL of 2 mM phenylalanine and 800 μL of enzymatic extract for phenylalanine ammonia-lyase assay. The production of cinnamic acid from phenylalanine was stopped by adding 100 μL of hydrochloric acid. Then, 2 ml of toluene was added and centrifuged at 5000 rpm. The upper layer containing trans-cinnamic acid was read at 290 nm by a spectrophotometer.

### The non-enzymatic antioxidant assays

Carotenoid, total phenol, and total flavonoid content in the leaves were assessed by different protocols.

### Carotenoid concentration assessment

Based on Lichtenthaler's method^25^, crushed fresh leaves were extracted in 80% (v/v) acetone and the light absorbance was read by a spectrophotometer at 470 nm. Acetone 80% was used as a control for the solution. Calculations were conducted using the following formula:

4 $${\text{Car }}\left( \upmu {{\text{g ml}}^{{ - {1}}} {\text{solution}}} \right) \, = \, \left( {{1}000{\text{ A47}}0 \, {-}{ 1}.{\text{82 Chl}}.{\text{a }}{-}{ 85}.0{\text{2 Chl}}.{\text{b}}} \right) \, /{ 198}$$

where A_470_ refers to the absorbance value of the corresponding wavelength. Carotenoid content was expressed according to the volume of the extract and the weight of the samples based on milligrams per gram of fresh weight (mg g^−1^ FW).

### Determination of total phenol

Leaf samples were crushed with 10 ml of 85% methanol. For the assay, 300 µl of the extract were combined with 1500 µl of diluted Folin reagent (1:10 ratio). After 5 min, 1200 µl of 7% sodium carbonate was added and after 90 min absorbance was read by spectrophotometer at 760 nm. Compared to the standard curve of Gallic acid, total phenolic content was expressed in micrograms of Gallic acid per dry weight^35^.

### Determination of total flavonoid

Based on method Kaijv^36^, 75 µl (5% w/v NaNO_2_), 0.15 ml AlCl_3_ (10% w/v) and 0.5 ml NaOH (1 M) distilled water were added to the leaf extract sample (0.25 ml), and the final volume reached 2.5 ml. After 5 min, the absorbance of the solution was read by the spectrophotometer at 507 nm.

### Proline concentration assay

Fresh leaves were dissolved in 10 ml of 3% sulfosalicylic acid solution and then centrifuged at 20,000*g* for 3 min. Also, 2 ml of supernatant was mixed with 2 ml of ninhydrin reagent and 2 ml of pure acetic acid for 1 h at 100 °C in a warm water bath. After the samples were cooled at room temperature, 4 ml of toluene was added to them. The colored upper layer containing toluene and proline was used to measure the l-proline concentration by a UV/vis spectrophotometer at 520 nm^37^.

### Statistical analysis

Before analysis of variance, the normal distribution and homology of variances were checked by statistical analysis software (SAS) using Shapiro–Wilk and Kolmogorov–Smirnov tests. The physiological and biochemical traits of leaves have two factors, including the season in three levels (spring, summer, and autumn) and sites in four levels (1, 2, 3, and 4) with three replications were analyzed in a completely randomized design. Analysis of variance by two-way ANOVA and a comparison of the means of all measured parameters was performed using the Duncan test at *P* < 0.05 by SPSS software version 24.

## Results

### Air pollution tolerance index

#### Total chlorophyll content

The results of total chlorophyll contents confirmed that *A. altissima* in spring at site 3 has the highest chlorophyll content (1.72 mg g^−1^ FW) compared to all evaluated sites. The chlorophyll contents in summer were 6.3 and 2.52 mg g^−1^ FW for sites 2 and 1, respectively. Moreover, the highest and lowest contents were 1.04 and 0.57 mg g^−1^ FW at sites 4 and 2 during the autumn. The chlorophyll content of *S. babylonica* at site 4 was noticeably higher than at other sites for all three seasons (*P* < 0.05). At sites 3 and 4, *M. alba* showed a noticeable increment in chlorophyll content in comparison to sites 1 and 2 (P < 0.05, Table 1).

**Table 1:** Comparing the effect of air pollution stress on the total chlorophyll concentration (fresh weight, FW) and relative water contents of *A. altissima*, *S. babylonica,* and *M. alba* trees.

Trees	Sites	Total chlorophyll contents (mg g^−1^ FW)			Relative water contents (%)			
		Spring	Summer	Autumn	Spring	Summer	Autumn	
*A. altissima*	1	1.39^op^ ± 0.005	2.52^j^ ± 0.02	1.05^t^ ± 0.01	50^ghi^ ± 0.03	64.82^d-h^ ± 0.13	66.4^bcd^ ± 0.10	
	2	1.26^qr^ ± 0.01	6.3^c^ ± 0.05	0.57^w^ ± 0.01	50.66^hij^ ± 0.14	70.77^a-g^ ± 0.06	79.25^abc^ ± 0.17	
	3	1.72^n^ ± 0.01	5.37^d^ ± 0.01	1.37^op^ ± 0.05	56.3^d-i^ ± 0.09	66.53^a-g^ ± 0.10	72.69^c-h^ ± 0.07	
	4	1.28^q^ ± 0.01	4.69^ g^ ± 0.05	1.04^t^ ± 0.02	50.66^hij^ ± 0.07	47.58^hij^ ± 0.01	62.4^c^ ± 0.02	
*S. babylonica*	1	1.06^t^ ± 0.02	3.31^i^ ± 0.01	1.35^qp^ ± 0.03	48.33^hij^ ± 0.03	63.86^c-h^ ± 0.08	65.69^b^ ± 0.16	
	2	1.27^q^ ± 0.008	3.26^i^ ± 0.005	1.9^ lm^ ± 0.02	85.9^c-h^ ± 0.19	48.55^hij^ ± 1.8	84.89^abc^ ± 0.85	
	3	1.7^n^ ± 0.008	3.72^ h^ ± 0.02	2.09^ k^ ± 0.01	34.33^ k^ ± 0.06	82.38^a-d^ ± 0.08	89.5^a^ ± 0.11	
	4	1.95 l ± 0.02	4.85^f^ ± 0.08	1.87^ lm^ ± 0.02	49^ij^ ± 0.38	84.4^cde^ ± 0.11	74.9^a-e^ ± 0.20	
*M. alba*	1	0.83^u^ ± 0.02	4.94^e^ ± 0.01	1.01^t^ ± 0.04	54^f-i^ ± 0.03	72.9^a-g^ ± 0.14	86.3^ab^ ± 0.45	
	2	0.89^u^ ± 0.006	5.35^d^ ± 0.02	1.08° ± 0.01	67^c-i^ ± 0.24	86.63^c-h^ ± 0.02	89.22^a^ ± 0.39	
	3	1.45° ± 0.02	6.4^b^ ± 0.09	1.17^rs^ ± 0.01	62.66^e-i^ ± 0.24	60.9^e-i^ ± 0.27	80.55^abc^ ± 0.27	
	4	1.83^ m^ ± 0.02	7.1^a^ ± 0.005	1.17^ s^ ± 0.08	63.3^d-i^ ± 0.27	81.87^a-d^ ± 0.05	76.17^a-e^ ± 0.31	

Three seasons of Spring, Summer, and Autumn at Sites 1 (Mirdamad Street), 2 (Mirza Babaei Street), 3 (Kargar Shomali Street), and 4 (Azadi Bus Station Street) Tehran. The means that have at least one common letter did not show a statistically significant difference (*P* < 0.05). Data are presented as mean ± SE.

#### Relative water content

As shown in Table 1, *A. altissima* showed no significant difference at all sites during the spring and autumn seasons. The lowest content was observed at site 4 (47.58%) in summer. Besides, we detected that *S. babylonica* had no significant changes in the spring and autumn seasons at sites 1, 2, and 4. In summer, the highest water content was observed at site 3 (4.85%). There was no significant difference (*P* > 0.05) in the relative water content of *M. alba* at all sites in the three seasons (Table 1).

#### pH

The leaf pH of *A. altissima* was 5.20 and 3.46, in spring at sites 3 and 4, respectively which are the highest and lowest pH values. Among all sites, trees at site 4 had high ascorbic acid concentration, whereas, site 2 had low value. Regardless of sites, the pH value concentrations measured for *A. altissima* increased with changing seasons from spring (4.76, site 1; 3.96, site 2; 3.46, site 4) and summer (5.4, site 1; 4.9, site 2; 5.13, site 4) to autumn (5.53, site 1; 5.46, site 2; 5.73, site 4). Besides, we detected pH values of 6.0 and 4.9 for *S. babylonica* leaves at sites 1 and 3, respectively. During the autumn and summer, when compared to all sites, site 1 had a high pH concentration. Based on the Table 2 data, the pH of *M. alba* leaves was in the range of 5.8 to 7.4, which was the highest measured value among all tree types.

**Table 2:** Comparison of the air pollution stress effect on the pH and Ascorbic acid concentration (fresh weight, FW) of *A. altissima*, *S. babylonica,* and *M. alba* trees.

Trees	Sites	pH			Ascorbic acid concentration (mg g^−1^ FW)		
		Spring	Summer	Autumn	Spring	Summer	Autumn
*A .altissima*	1	4.76° ± 0.12	5.4^i-o^ ± 0.11	5.53^ h-m^ ± 0.20	5.2^ijk^ ± 0.20	4.55^klm^ ± 0.14	7.01^ g^ ± 0.06
	2	3.96^p^ ± 0.14	4.9^no^ ± 0.10	5.46^ h-n^ ± 0.08	3.66^nop^ ± 0.14	3.91^ m-p^ ± 0.14	5.72^hi^ ± 0.17
	3	5.20^ k-o^ ± 0.11	5.55^ h-m^ ± 0.20	5.4^i-o^ ± 0.05	6.71 g ± 0.33	5.4^i^ ± 0.22	9.83^ cd^ ± 0.12
	4	3.46^q^ ± 0.26	5.13^ l-o^ ± 0.08	5.73^ g-k^ ± 0.23	7.8^f^ ± 0.10	6.67^ g^ ± 0.22	11.04^b^ ± 0.07
*S. babylonica*	1	5.36^i-o^ ± 0.18	5.7^ h-l^ ± 0.30	6^d-h^ ± 0.5	3.89^ m-p^ ± 0.20	4.32l^mn^ ± 0.31	6.79^ g^ ± 0.05
	2	5.06^mno^ ± 0.12	5.33^i-o^ ± 0.03	5.86^e-i^ ± 0.34	3.37^p^ ± 0.26	3.66^nop^ ± 0.18	5.58^i^ ± 0.15
	3	4.90^no^ ± 0.10	5.53^ h-m^ ± 0.23	5.43^ h-m^ ± 0.06	4.63^j-m^ ± 0.22	5.31^ij^ ± 0.12	8.56^e^ ± 0.06
	4	5.53^ h-m^ ± 0.13	5.23^j-o^ ± 0.12	5.53^ h-m^ ± 0.03	5.54^i^ ± 0.42	6.34^gh^ ± 0.16	9.34^ cd^ ± 0.10
*M. alba*	1	6.93^ab^ ± 0.37	6.53^bcd^ ± 0.14	6.26^c-g^ ± 0.21	4.69^jkl^ ± 0.61	4.12^mno^ ± 0.09	9.25^d^ ± 0.10
	2	5.80^f-j^ ± 0.6	6.53^bcd^ ± 0.14	5.83^e-i^ ± 0.16	4.52^klm^ ± 0.44	3.49^op^ ± 0.09	8.09^ef^ ± 0.19
	3	6.33^cde^ ± 0.08	7.4^a^ ± 0.05	7.26^a^ ± 0.12	7.75^f^ ± 0.29	6.33^gh^ ± 0.08	9.90^c^ ± 0.10
	4	6.60^bc^ ± 0.15	6.7^bc^ ± 0.20	6.36^cde^ ± 0.13	7.75^e^ ± 0.20	7.8^f^ ± 0.41	13.04^a^ ± 0.13

Three seasons of Spring, Summer, and Autumn at Sites 1 (Mirdamad Street), 2 (Mirza Babaei Street), 3 (Kargar Shomali Street), and 4 (Azadi Bus Station Street) Tehran. The means that have at least one common letter did not show a statistically significant difference (*P* < 0.05). Data are presented as mean ± SE.

#### Ascorbic acid

The next section of Table 2 illustrates the detected ascorbic acid concentration values. The ascorbic acid concentration varies between 3.37 to 13.04 mg g^−1^. Interestingly, the measured ascorbic acid content value in autumn was more than in summer and spring. In detail, for A. *altissima*, 7.8, 6.67, and 11.04 mg g^−1^ ascorbic acid concentrations were reported at site 4. Also, *S. babylonica* had 5.54, 6.34, and 9.34 mg g^−1^ ascorbic acid concentrations. *M. alba* had 7.75, 7.8, and 13.04 mg g^−1^ concentrations.

### Air pollution tolerance index

**Figure 2:**
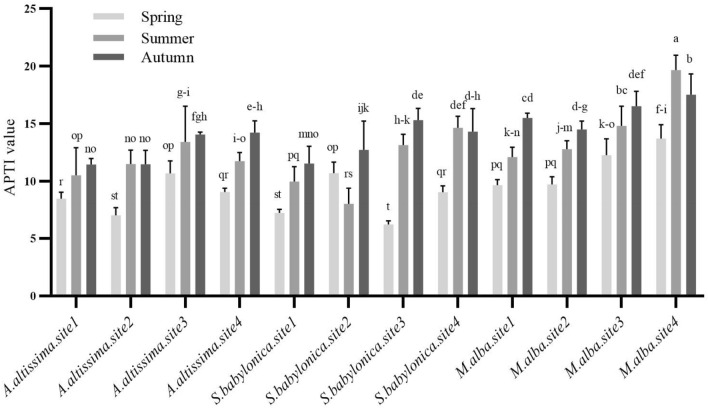
APTI values of *A. altissima, S. babylonica*, and *M. alba* trees in Spring, Summer, and Autumn seasons at sites 1 (Mirdamad Street), 2 (Mirza Babaei Street), 3 (Kargar Shomali Street), and 4 (Azadi Bus Station Street) of Tehran. The means that have at least one common letter did not show a statistically significant difference (*P* < 0.05). Data are presented as mean ± SE.

Compared to the spring and summer, *A. altissima* had high APTI values in autumn regardless of examined sites. *A. altissima* had 10.69 and 7.01 APTI values at sites 3 and 2, respectively. In summer, the highest APTI value of *A. altissima* was observed at site 3 (13.43). Further, the APTI values at sites 3 and 4 were significantly higher than at sites 1 and 2 in autumn (P < 0.05). On the other hand, the APTI value of *S. babylonica* peaked at 10.72 in spring at site 2, whereas the minimal APTI value, 6.21, was at site 3. In summer, the top and lowest APTI values at sites 4 and 2 were 14.65 and 8 for *S. babylonica*, respectively. In autumn sites 3 and 4 showed noticeable changes and had higher APTI values than sites 1 and 2 (*P* < 0.05). Finally, the APTI value of *M. alba* in the spring, summer and autumn seasons at sites 3 and 4 were significantly higher than sites 1 and 2 (*P* < 0.05). *M. alba* had the highest APTI value (14.08) among all tree types (Fig. 2).

### The enzymatic antioxidants

Among the investigated sites, the uppermost catalase activity was reported for *A. altissima* in summer at site 3 (0.95 ∆OD min^−1^; *P* < 0.05). While the highest catalase activity was observed in autumn, site 3, the lowest catalase activity was at sites 1 and 4. The catalase activity of *S. babylonica* in the summer and autumn seasons at sites 3 and 2 were significantly higher than at sites 1 and 4 (P < 0.05). Moreover, compared to sites 1 and 2, the catalase activity of *M. alba* was significantly high (*P* < 0.05) in the summer and autumn seasons at sites 3 and 4. The highest and lowest catalase activity of *M. alba* was observed at sites 3 and 2, respectively (Table 3). *S. babylonica* had significantly higher ascorbate peroxidase activity in summer, at sites 1 and 3 than at sites 2 and 4. In autumn, minimal ascorbate peroxidase activity was observed at site 2 (0.11 ∆OD min^−1^). Contrary to the summer, ascorbate peroxidase activity of *M. alba* in autumn at sites 3 and 4, was significantly higher than at sites 1 and 2 (*P* < 0.05). Notably, the phenylalanine ammonia-lyase activity of *A. altissima*, *S. babylonica*, and *M. alba* trees were significantly higher in the summer and autumn seasons at sites 3 and 4 than at sites 1 and 2 (*P* < 0.05; Table 3).

**Table 3:** Comparing the effect of air pollution stress on the catalase, ascorbate peroxidase and PAL activities of *A. altissima*, *S. babylonica*, and *M. alba* trees.

Trees	Sites	Catalase activity (∆OD min^−1^)		Ascorbate peroxidase activity (∆OD min^−1^)		PAL activity (μΜ cinnamic acid min^−1^)	
		Summer	Autumn	Summer	Autumn	Summer	Autumn
*A. altissima*	1	0.45^ fg^ ± 0.01	0.15^j-l^ ± 0.01	0.20^d-h^ ± 0.005	0.34^bc^ ± 0.02	1.29^p^ ± 0.38	2.96^i^ ± 0.3
	2	0.44^ fg^ ± 0.03	0.49^e-g^ ± 0.04	0.34^bc^ ± 0.03	0.23^c-g^ ± 0.21	0.92^q^ ± 0.48	1.92^ m^ ± 0.28
	3	0.95^ab^ ± 0.01	0.62^c-e^ ± 0.01	0.2^d-h^ ± 0.02	0.5^a^ ± 0.01	1.76^n^ ± 0.23	3.8^e^ ± 0.16
	4	0.45^ fg^ ± 0.08	0.14^j-l^ ± 0.01	0.35^bc^ ± 0.01	0.04^i^ ± 0.005	1.97^ m^ ± 0.45	4.52^c^ ± 1.1
*S. babylonica*	1	0.12^j-l^ ± 0.01	0.28^ h-j^ ± 0.05	0.25^c-f^ ± 0.01	0.3^b-d^ ± 0.003	0.89^q^ ± 0.21	3.22^b^ ± 0.74
	2	0.45^ fg^ ± 0.01	0.33^ g-i^ ± 0.01	0.03^i^ ± 0.02	0.11^f-i^ ± 0.02	0.64^p^ ± 0.1	2.5^ k^ ± 0.63
	3	0.53^d-f^ ± 0.03	0.65^ cd^ ± 0.01	0.31^b-d^ ± 0.01	0.27^c-e^ ± 0.02	1.24^p^ ± 0.57	3.61^f^ ± 1.01
	4	0.12^j-l^ ± 0.01	0.21^i-k^ ± 0.03	0.05^i^ ± 0.003	0.35^bc^ ± 0.01	1.55° ± 0.16	4.15^d^ ± 0.19
*M. alba*	1	0.71^c^ ± 0.003	0.87^ab^ ± 0.006	0.06^hi^ ± 0.02	0.02^i^ ± 0.02	2.57^ k^ ± 0.55	4.12^d^ ± 0.56
	2	0.34^ g-i^ ± 0.003	0.43^f-h^ ± 0.01	0.06^hi^ ± 0.10	0.07^hi^ ± 0.03	2.42^i^ ± 0.66	2.82^j^ ± 0.86
	3	1.05^a^ ± 0. 03	1.02^ab^ ± 0.01	0.16^e-i^ ± 0.01	0.25^c-f^ ± 0.04	2.96^i^ ± 0.82	6.41^b^ ± 1.7
	4	0.89^ab^ ± 0. 02	0.96^b^ ± 0.02	0.1^ g-i^ ± 0.02	0.43^ab^ ± 0.02	3.5^ g^ ± 1.1	8.17^a^ ± 1.3

Two seasons of Summer and Autumn at Sites 1 (Mirdamad Street), 2 (Mirza Babaei Street), 3 (Kargar Shomali Street), and 4 (Azadi Bus Station Street) Tehran. The means that have at least one common letter did not show a statistically significant difference (*P* < 0.05). Data are presented as mean ± SE.

*PAL* phenyalanine amonia lyase.

### The non-enzymatic antioxidants

**Figure 3:**
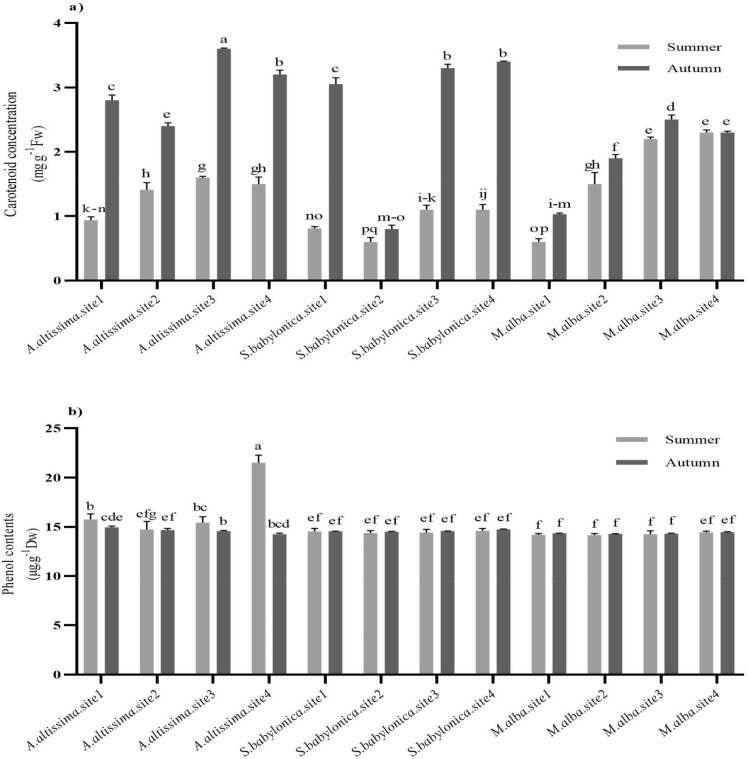
Carotenoid concentrations (**a**) and phenol contents (**b**) of *A. altissima, S. babylonica*, and *M. alba* trees in spring, summer, and autumn at sites 1 (Mirdamad Street), 2 (Mirza Babaei Street), 3 (Kargar Shomali Street), and 4 (Azadi Bus Station Street) of Tehran. The means that have at least one common letter did not show a statistically significant difference (*P* < 0.05). Data are presented as mean ± SE.

The carotenoid concentration of *A. altissima, S. babylonica,* and *M. alba* were significantly high in summer and autumn at sites 3 and 4 in comparison to sites 1 and 2 (*P* < 0.05; Fig. 3a). Among four evaluated sites, the total phenol content of *M. alba* and *S. babylonica* trees in summer and autumn did not change significantly (*P* > 0.05). *A. altissima* in summer at site 4 had maximum total phenol content between the other sites (21.51 µg g^−1^ Dw; Fig. 3b).

**Figure 4:**
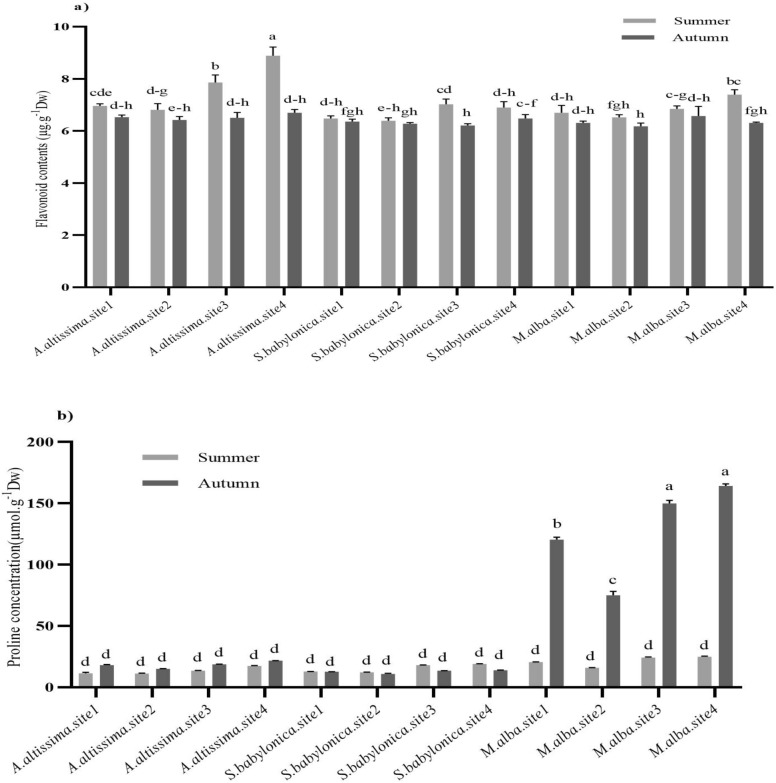
Flavonoid contents (**a**) and proline concentration (**b**) of *A. altissima, S. babylonica*, and *M. alba* trees in spring, summer, and autumn at sites 1 (Mirdamad Street), 2 (Mirza Babaei Street), 3 (Kargar Shomali Street), and 4 (Azadi Bus Station Street) of Tehran. The means that have at least one common letter did not show a statistically significant difference (*P* < 0.05). Data are presented as mean ± SE.

Flavonoid contents of *M. alba* and *A. altissima* were significantly higher in summer at sites 3 and 4 than at sites 1 and 2. In summer and autumn, the flavonoid content of *S. babylonica* did not significantly change at four sites. In autumn, there was no significant difference in flavonoid content of *M. alba* at four sites (Fig. 4a). Further, regarding the proline concentration of *A. altissima* and *S. babylonica* trees, there was no significant difference among the four sites in the summer and autumn seasons (*P* > 0.05). Proline concentration of *M. alba* in the summer season was not significantly changed among the four sites. In autumn, sites 3 and 4 had significantly (*P* < 0.05) higher proline than sites 1 and 2 (Fig. 4b).

## Discussions

Trees act as biomonitoring sensors and a remedy to urban air pollution by purifying PM_2.5_. Choosing suitable plant species is essential for designing urban greening to mitigate air pollution, and it is based on the plant sensitivity and tolerance to air pollution levels^38^. Although *Morus alba*, *Ailanthus altissima*, and *Salix babylonica* trees are broadleaf plants, they have an important role in the urban ecosystem of Tehran as these trees are distributed extensively on highways and urban green spaces in different areas of the city. Therefore, *Morus alba*, *Ailanthus altissima*, and *Salix babylonica* were selected because they influence directly and indirectly the life of 12 million people in Tehran. Importantly, their leaves are available until the beginning of the winter season, which allow us to measure the APTI values in different seasons. On the other hand, for the first time, this study aimed to evaluate the role of physiological and biochemical responses of Tehran's high distribution trees to air pollution.

Our result indicates that APTI values increased significantly during polluted seasons of the year and at sites in the city. Our findings reveal that *M. alba* with an APTI of 14.08 was intermediate tolerance to air pollution in Tehran. Likewise, *Thespesia populnea* and *Pongamia pinnata* trees exhibited the highest (APTI > 13) and were tolerant to air pollution when 25 tree species were evaluated for their air pollution tolerance in various urban locations of an Indian city. In addition, they performed admirably in controlling urban pollution levels^39^.

The *A. altissima* and *S. babylonica* trees, with APTI of 11.15 and 11.08, were sensitive, suggesting that these trees can be used for air pollution monitoring. Similarly, Anake et al., who evaluated the air pollution tolerance of six mature tree species in different parts of a Nigerian city, discovered that all trees with an APTI < 11 were sensitive to air pollution and might be used as bioindicators for the air pollution of this city^31^.

Proline regulates osmosis, protects photosynthetic components and proteins and cell membranes from osmotic stress, and increases antioxidants for free radical reduction^40^. Regarding proline concentration, whilst *S. babylonica* and *A. altissima* trees were not significantly different at polluted sites and seasons, *M. alba* had a higher concentration at the polluted sites. Hence, it appears that high proline concentration protected M. *alba* from oxidative stress damage and was accompanied by more tolerance to air pollution compared to other trees. These findings are consistent with the results of Rai et al.^41^ in India, who found that *Magnifera indica* L. compared to *Thevetia neriifolia* L. and *Psidium guajava* L. had the highest proline concentration in leaf organs.

Ascorbic acid is not only an antioxidant that protects plants from environmental threats like air pollution, but it is also a powerful reducing agent that activates many defenses and physiological mechanisms in the plant^42,43^. Besides, its reducing power is dependent on the pH of the cell and is active at a higher pH^44^. Our observation revealed that all trees had high ascorbic acid in pollution sites and seasons of the year for tolerance of abiotic stress. Some plants, such as *Taraxacum officinale*, increase their ascorbic acid levels in areas with high air pollution stress^45^.

To gain insights into the influence of pH, we analyzed the pH of leaf extracts of trees at all the mentioned sites and seasons. Our data revealed that almost all trees in spring had a low pH, whereas, in the autumn season, the pH was near neutral. Different pollutants could be inserted into tree cells and diminish the pH of their conditions (acidic pH), whereupon *A. altissima* and *S. babylonica* with lower pH may be sensitive to air pollution. Compared to other trees, *M. alba* approximately had neutral pH, showing that it was more tolerant to pollution. A similar result was reached by Joshi et al.^46^ that low leaf pH has a direct relationship with leaf susceptibility of different plant species to air pollution. Penetration of pollutants induces acidification of the intracellular pH, which is more common in sensitive species. rees in polluted seasons and locations with increased cell pH attempted to convert different hexoses carbohydrates to ascorbic acid. Thus, these mechanisms are different tolerance ways for trees to combat oxidative stress^47^.

We showed that trees had the highest chlorophyll concentration in summer. In summer and following autumn, air pollution levels were high and trees were tolerant as their chlorophyll levels increased. Besides, they had higher chlorophyll in pollution sites compared to other low pollution sites. In some plants, the dry and wet weight of the leaves and consequently their total chlorophyll increase in leaves that are exposed to air pollution stress. This increase can be a sign of their greater tolerance and resistance to this stress^48,49^. The chlorophyll content of the leaves of *Taraxacum officinale*, *Plantago lanceolata*, *Betula pendula*, and *Robinia pseudoacacia* L. is heavily influenced by pollution sources such as heavy traffic, polluted air, and industrial activities^45^.

The water content of trees changes in different seasons to maintain water potential for cells. *M. alba* trees maintain relatively higher water content than other trees in different sites and seasons. Other trees had irregular relative water content in pollution conditions, which means they were sensitive to osmotic stress. Therefore, maintaining the cell water content of studied trees was one of the indicators of tree tolerance to air pollution. When plants are confronted with non-biological stress such as air pollution, their water content increases, and physiological equilibrium is maintained, to resist the stress^50,51^. Our observation indicated that trees at pollution sites and seasons increased their intracellular water content, which led to the well-conducting of different physiological mechanisms. Thus, optimum water content is essential for cells when they are subjected to air pollution^52^.

It is known that enzymatic activity is high in polluted sites and can be termed a genetic adaptation to reduce the toxic level of hydrogen peroxide and avoid any damage to plant tissue^53^. Our results showed that examined trees had higher catalase, ascorbate peroxidase, and PAL activities in polluted sites and times of the year. As a sensitive indicator of plant defense changes against environmental stresses, the phenylalanine ammonium lyase enzyme is the initiator of the phenylpropanoid pathway, which is a major biosynthetic pathway of secondary metabolites in plant cells^54,55^. In our study, when trees were subjected to pollution, the phenylalanine ammonium enzyme was activated and reached its highest level within cells. By subjecting cells to oxidative stress, different metabolites that can respond to stress are activated through signaling pathways such as phenylpropanoid. So, this enzyme had an important function in the tolerance of cell components^56^.

In this study, all of the trees activate this pathway by phenylalanine ammonium enzymes, which is an important defense mechanism against abiotic stress. This is in line with the previous work by Rai^57^, which shows that roadside plants respond to air pollution in Aizawl City, India by increasing their catalase and peroxidase activities.

Phenolic metabolites are induced by stress and accumulate in tissues due to biological and non-biological stresses^58^. They may participate in the removal of reactive oxygen species through antioxidant enzymes or directly chelate metals. By reducing or inhibiting lipid auto-oxidation or decomposing peroxides, they are essential antioxidants to protect against the proliferation and progression of the oxidation chain and protect against reactive oxygen species^59,60^. Although phenols and flavonoids were present in plants as byproducts of related pathways to the phenylalanine ammonia-lyase enzyme, their concentrations could be increased under oxidative stress. In contrast to the *M. alba* and *S. babylonica* trees, in which the phenolic and flavonoid activities were unchanged, in *A. altissima* an increase was recorded. Under conditions of high air pollution, the genetic responses of these trees activate the phenylpropanoid pathway. Thus, different responses of trees and pollution conditions in the Tehran metropolis indicated that secondary metabolites such as total phenol and total flavonoid were different in our study. Our results were broadly in line with other results that oak species such as *Quercus pubescens* L*.* and *Quercus ilex* L. unchanged their total phenols because they have a superior ability to counteract oxidative stress^56^.

Carotenoid is one of the antioxidants that plays an important function in the tolerance of oxidative stresses^61^. Plants appear to be trying to protect chlorophyll from damage by increasing the concentration of carotenoids^62^. When cells are subjected to abiotic stress, the cell membrane is damaged and the level of thylakoid and chlorophyll in the chloroplast is diminished^63^.

Carotenoids are pigments whose biosynthesis pathway is similar to chlorophyll which protects them from photooxidation and oxidative stress. Therefore, the concentrations of chlorophyll and carotenoids were related to each other in the chloroplast structure^64^. Our findings demonstrated that carotenoid concentrations increased in different seasons in polluted sites to reduce photooxidative stress effects on chlorophyll components. This is consistent with a finding by Pellegrini et al.^56^, that showed where *Quercus ilex* L. trees are affected by different stresses, carotenoids concentrations increased with the diminishing of peroxidation free radicals from these stresses.

Tree species are the main components of urban greening, and different factors influence their survival in cities. This research measured the tolerance mechanisms of trees to environmental stress such as air pollution^65^. There were certain limitations, including the sampling of PM_2.5_ in the area surrounding the tree canopy and the evaluation of hydraulic pathways in the wood and phloem vessels of trees. These measurements are valuable for future research. In addition, it is suggested that managers and designers of urban ecosystems take into account the amount of evapotranspiration and the water requirements of trees in future urban greening in water-stressed cities such as Tehran.

## Conclusions

To develop suitable green infrastructure to remediate and manage air pollution in Tehran, Iran, the tolerance of *Morus alba*, *Ailanthus altissima*, and *Salix babylonica* were evaluated in four regions of this megacity at different times of the year based on the APTI values. This research shows that *Morus alba* with a high APTI value had a medium-to-high tolerance to air pollution, while *Ailanthus altissima* and *Salix babylonica* with a low APTI value were sensitive to air pollution. All trees, especially M. alba in autumn and sites 3 and 4, had higher APTI values than other seasons and sites of the city. The PAL activity and carotenoid concentration of trees were significantly higher in summer and autumn at sites 3 and 4 than at sites 1 and 2. The total phenol content of trees did not change significantly at the four evaluated sites. The *M. alba*, unlike other trees that did not differ in proline concentration, had the highest proline in autumn and at sites 3 and 4. Considering these findings, *Morus alba* is suggested to be utilized as the main tree in designing resistant urban greening to mitigate air pollutant risks. Overall, our observations revealed that selecting suitable plants, which can be a remedy against air pollution and act as an indicator for measuring air quality, is an innovative and emerging strategy for future environmental management of megacities.

## Supplementary Information

Supplementary Information.

## Data Availability

All data generated or analyzed during this study are included in this published article (and its supplementary information files).

## References

[1] Künzli N (2000). Public-health impact of outdoor and traffic-related air pollution: A European assessment. Lancet.

[2] Dehghani M, Anushiravani A, Hashemi H, Shamsedini N (2014). Survey on air pollution and cardiopulmonary mortality in shiraz from 2011 to 2012: An analytical-descriptive study. Int. J. Prevent. Med..

[3] Carvalho-Oliveira R (2017). Effectiveness of traffic-related elements in tree bark and pollen abortion rates for assessing air pollution exposure on respiratory mortality rates. Environ. Int..

[4] Alahabadi A (2017). A comparative study on capability of different tree species in accumulating heavy metals from soil and ambient air. Chemosphere.

[5] Achakzai K (2017). Air pollution tolerance index of plants around brick kilns in Rawalpindi, Pakistan. J. Environ. Manag..

[6] Jonak, C., Nakagami, H. & Hirt, H. Heavy metal stress. Activation of distinct mitogen-activated protein kinase pathways by copper and cadmium. *Plant Physiol.* **136**, 3276–3283 (2004). 10.1104/pp.104.045724 PMC523386 15448198

[7] Shahid M (2014). Heavy-metal-induced reactive oxygen species: Phytotoxicity and physicochemical changes in plants. Rev. Environ. Contam. Toxicol..

[8] Tausz M, Grulke NE, Wieser G (2007). Defense and avoidance of ozone under global change. Environ. Pollut..

[9] Gill SS, Tuteja N (2010). Reactive oxygen species and antioxidant machinery in abiotic stress tolerance in crop plants. Plant Physiol. Biochem..

[10] Foyer CH, Shigeoka S (2011). Understanding oxidative stress and antioxidant functions to enhance photosynthesis. Plant Physiol..

[11] Krishnaveni M (2013). Air pollution tolerance index and antioxidant activity of *Parthenium hysterophorus*. J. Pharm. Res..

[12] Ashraf M, Foolad M (2007). Roles of glycine betaine and proline in improving plant abiotic stress resistance. Environ. Exp. Bot..

[13] Islam MM (2009). Exogenous proline and glycinebetaine increase antioxidant enzyme activities and confer tolerance to cadmium stress in cultured tobacco cells. J. Plant Physiol..

[14] Singh, M., Singh, V. P., Dubey, G. & Prasad, S. M. Exogenous proline application ameliorates toxic effects of arsenate in *Solanum melongena* L. seedlings. *Ecotoxicol. Environ. Saf.* **117**, 164–173 (2015). 10.1016/j.ecoenv.2015.03.021 25881134

[15] Aggarwal, M. *et al.* Exogenous proline application reduces phytotoxic effects of selenium by minimising oxidative stress and improves growth in bean (*Phaseolus vulgaris* L.) seedlings. *Biol. Trace Elem. Res.* **140**, 354–367 (2011). 10.1007/s12011-010-8699-9 20455031

[16] Alotaibi MD (2020). Assessing the response of five tree species to air pollution in Riyadh City, Saudi Arabia, for potential green belt application. Environ. Sci. Pollut. Res..

[17] Manjunath B, Reddy J (2019). Comparative evaluation of air pollution tolerance of plants from polluted and non-polluted regions of Bengaluru. J. Appl. Biol. Biotechnol..

[18] Sanaeirad, H., Majd, A., Abbaspour, H. & Peyvandi, M. The effect of air pollution on proline and protein content and activity of nitrate reductase enzyme in *Laurus nobilis* L. plants. *J. Mol. Biol. Res.* **7**, 99–105 (2017).

[19] Khosropour E (2019). Response of *Platanus orientalis* leaves to urban pollution by heavy metals. J. For. Res..

[20] Motlagh SHB, Pons O, Hosseini SA (2021). Sustainability model to assess the suitability of green roof alternatives for urban air pollution reduction applied in Tehran. Build. Environ..

[21] Sadeghi, S. M. M., Van Stan II, J. T., Pypker, T. G. & Friesen, J. Canopy hydrometeorological dynamics across a chronosequence of a globally invasive species, *Ailanthus altissima* (Mill., tree of heaven). *Agric. For. Meteorol.* **240**, 10–17 (2017).

[22] Faridi, S. *et al.* Long-term trends and health impact of PM_2.5_ and O_3_ in Tehran, Iran, 2006–2015. *Environ. Int.* **114**, 37–49 (2018). 10.1016/j.envint.2018.02.026 29477017

[23] Heshmatol Vaezin, S. M. *et al.* The effectiveness of urban trees in reducing airborne particulate matter by dry deposition in Tehran, Iran. *Environ. Monit. Assess.* **193**, 1–14 (2021). 10.1007/s10661-021-09616-8 34821985

[24] Ghahraman, A. Colorful Flora of Iran. The Research Institute of Forest and Pastures, Tehran. Implication to Biodiversity Conservation. *SINET Ethiop. J. Sci.* **30**, 1–12 (1979).

[25] Lichtenthaler, H.K, Chlorophylls and carotenoids, the pigments of photosynthetic biomembranes. in *Methods Enzymol*ogy (Douce, R., Packer, L. eds.). Vol. 148. 350–382 (Academic Press Inc., 1987).

[26] Liu Y-J, Ding H (2008). Variation in air pollution tolerance index of plants near a steel factory: Implication for landscape-plant species selection for industrial areas. WSEAS Trans. Environ. Dev..

[27] Zhang J (2016). Analysis of effects of a new environmental pollutant, bisphenol A, on antioxidant systems in soybean roots at different growth stages. Sci. Rep..

[28] Kampfenkel K, Vanmontagu M, Inzé D (1995). Extraction and determination of ascorbate and dehydroascorbate from plant tissue. Anal. Biochem..

[29] Singh S, Rao D, Agrawal M, Pandey J, Naryan D (1991). Air pollution tolerance index of plants. J. Environ. Manag..

[30] Singh S, Rao D (1983). Evaluation of plants for their tolerance to air pollution. Proc. Sympos. Air Pollut. Control.

[31] Anake WU, Eimanehi JE, Omonhinmin CA (2019). Evaluation of air pollution tolerance index and anticipated performance index of selected plant species. Indones. J. Chem..

[32] Dhindsa R, Plumb-Dhindsa P, Thorpe TA (1981). Leaf senescence: Correlated with increased levels of membrane permeability and lipid peroxidation, and decreased levels of superoxide dismutase and catalase. J. Exp. Bot..

[33] Nakano Y, Asada K (1987). Purification of ascorbate peroxidase in spinach chloroplasts; its inactivation in ascorbate-depleted medium and reactivation by monodehydroascorbate radical. Plant Cell Physiol..

[34] D'Cunha GB, Satyanarayan V, Nair PM (1996). Stabilization of phenylalanine ammonia lyase containing *Rhodotorula glutinis* cells for the continuous synthesis of l-phenylalanine methyl ester/96/. Enzyme Microb. Technol..

[35] Singleton VL, Rossi JA (1965). Colorimetry of total phenolics with phosphomolybdic-phosphotungstic acid reagents. Am. J. Enol. Vitic..

[36] Kaijv M, Sheng L, Chao C (2006). Antioxidation of flavonoids of green rhizome. Food Sci..

[37] Bates L, Waldren R, Teare I (1973). Rapid determination of free proline for water-stress studies. Plant Soil.

[38] Zhang P-Q (2016). Pollution resistance assessment of existing landscape plants on Beijing streets based on air pollution tolerance index method. Ecotoxicol. Environ. Saf..

[39] Balasubramanian A, Prasath C, Gobalakrishnan K, Radhakrishnan S (2018). Air pollution tolerance index (APTI) assessment in tree species of Coimbatore urban city, Tamil Nadu, India. Int. J. Environ. Clim. Change.

[40] Bacelar EA, Moutinho-Pereira JM, Gonçalves BC, Lopes JI, Correia CM (2009). Physiological responses of different olive genotypes to drought conditions. Acta Physiol. Plant.

[41] Rai PK (2016). Biodiversity of roadside plants and their response to air pollution in an Indo-Burma hotspot region: Implications for urban ecosystem restoration. J. Asia-Pac. Biodivers..

[42] Keller T, Schwager H (1977). Air pollution and ascorbic acid. Eur. J. For. Pathol..

[43] Lima JS, Fernandes E, Fawcett W (2000). Mangifera indica and Phaseolus vulgaris in the bioindication of air pollution in Bahia, Brazil. Ecotoxicol. Environ. Saf..

[44] Agbaire P, Esiefarienrhe E (2009). Air pollution tolerance indices (APTI) of some plants around Otorogun Gas Plant in Delta State, Nigeria. J. Appl. Sci. Environ. Manag..

[45] Nadgórska-Socha A, Kandziora-Ciupa M, Trzęsicki M, Barczyk G (2017). Air pollution tolerance index and heavy metal bioaccumulation in selected plant species from urban biotopes. Chemosphere.

[46] Joshi N, Chauhan A, Joshi P (2009). Impact of industrial air pollutants on some biochemical parameters and yield in wheat and mustard plants. Environmentalist.

[47] Dadkhah-Aghdash, H. *et al.* Variation in Brant’s oak (*Quercus brantii* Lindl.) leaf traits in response to pollution from a gas refinery in semiarid forests of western Iran. *Environ. Sci. Pollut. Res.* **29**, 10366–10379 (2022). 10.1007/s11356-021-16270-7 34519983

[48] Seyyednejad S, Niknejad M, Koochak H (2011). A review of some different effects of air pollution on plants. Res. J. Environ. Sci..

[49] Singh, S. & Verma, A. *Environmental Bioremediation Technologies*. 293–314 (Springer, 2007).

[50] Verma S, Dubey R (2003). Lead toxicity induces lipid peroxidation and alters the activities of antioxidant enzymes in growing rice plants. Plant Sci..

[51] Rai PK, Panda LL, Chutia BM, Singh MM (2013). Comparative assessment of air pollution tolerance index (APTI) in the industrial (Rourkela) and non industrial area (Aizawl) of India: An ecomanagement approach. Afr. J. Environ. Sci. Technol..

[52] Prajapati SK, Tripathi B (2008). Seasonal variation of leaf dust accumulation and pigment content in plant species exposed to urban particulates pollution. J. Environ. Qual..

[53] Bansal P, Verma S, Srivastava A (2016). Biomonitoring of air pollution using antioxidative enzyme system in two genera of family Pottiaceae (Bryophyta). Environ. Pollut..

[54] Boudet A-M (2007). Evolution and current status of research in phenolic compounds. Phytochemistry.

[55] Vogt T (2010). Phenylpropanoid biosynthesis. Mol. Plant.

[56] Pellegrini E (2019). Antioxidative responses of three oak species under ozone and water stress conditions. Sci. Total Environ..

[57] Rai PK (2016). Impacts of particulate matter pollution on plants: Implications for environmental biomonitoring. Ecotoxicol. Environ. Saf..

[58] Nayak R, Biswal D, Sett R (2013). Biochemical changes in some deciduous tree species around Talcher thermal power station, Odisha, India. J. Environ. Biol..

[59] Kovacik J, Backor M (2007). Phenylalanine ammonia-lyase and phenolic compounds in chamomile tolerance to cadmium and copper excess. Water Air Soil Pollut..

[60] Egert M, Tevini M (2002). Influence of drought on some physiological parameters symptomatic for oxidative stress in leaves of chives (*Allium schoenoprasum*). Environ. Exp. Bot..

[61] Havaux M, Eymery F, Porfirova S, Rey P, Dörmann P (2005). Vitamin E protects against photoinhibition and photooxidative stress in *Arabidopsis thaliana*. Plant Cell.

[62] Jaleel CA (2009). Antioxidant defense responses: Physiological plasticity in higher plants under abiotic constraints. Acta Physiol. Plant..

[63] Qadir SU, Raja V, Siddiqui WA (2016). Morphological and biochemical changes in *Azadirachta indica* from coal combustion fly ash dumping site from a thermal power plant in Delhi, India. Ecotoxicol. Environ. Saf..

[64] Assadi A, Pirbalouti AG, Malekpoor F, Teimori N, Assadi L (2011). Impact of air pollution on physiological and morphological characteristics of *Eucalyptus camaldulensis*. J. Food Agric. Environ..

[65] Yazdanpanahrostami, A. & Rasouli, K. Simulation of Tehran air pollution using artificial neural networks. in *World Environmental and Water Resources Congress 2009: Great Rivers*. 1–11 (2009).

