# Supplementary material for: Detection of urban trees sensitivity to air pollution using physiological and biochemical leaf traits in Tehran, Iran
Source: Sci Rep. 2022 Sep 13;12:15398. doi: 10.1038/s41598-022-19865-3 (PMC9470701; doi:10.1038/s41598-022-19865-3)
Supplement: Supplementary file 1 — Supplementary Information. [file 41598_2022_19865_MOESM1_ESM.docx]

**Supplementary information**

**Detection of urban trees sensitivity to air pollution using physiological and biochemical leaf traits in Tehran, Iran**

Hamed Dadkhah-Aghdash^1*^, Milad Rasouli^2, 3^, Kabir Rasouli^4^, Azam Salimi^5^

| **Trees APTI Total chlorophyll (mg.g^-1^FW)** |
| --- |
| *M. alba* 14.08 2.76  *A. altissima* 11.15 2.37  *S. babylonica* 11.08 2.36 |
|  |

**Table S1.** Comparison of the APTI value and total chlorophyll of the trees of *M. alba*, *A. altissima,* and *S. babylonica*

| **Table S2.** The Comparison of the effect of air pollution stress on the Phenol, flavonoid and carotenoid contents and Proline concentration of the trees of *M. alba*, *A. altissima* and *S. babylonica*. | | | | | | | | | | | | | | | | | | | | | | | | | | | |  |  |  |  |
| --- | --- | --- | --- | --- | --- | --- | --- | --- | --- | --- | --- | --- | --- | --- | --- | --- | --- | --- | --- | --- | --- | --- | --- | --- | --- | --- | --- | --- | --- | --- | --- |
| ***Treatment*** | **Phenol contents**  **(µg.g^-1^Dw)** | | **Flavonoid contents**  **(µg.g^-1^Dw)** | | | | | **Carotenoid concentration**  **(mg.g^-1^Fw)** | | | | | | | | | | **Proline concentration**  **(µmol.g^1^Dw^-1^)** | | | | | | | | | |  |  |  |  |
|  | **Summer** | **Autumn** | **Summer** | | **Autumn** | | | **Summer** | | | | | **Autumn** | | | | | **Summer** | | | | | **Autumn** | | | | |  |  |  |  |
|  | Mean ± SE | Mean ± SE | Mean ± SE | | Mean ± SE | | | | Mean ± SE | | | | | Mean ± SE | | | | | Mean ± SE | | | | | Mean ± SE | | | | |  |  |  |
| *A.altissima*.site1 | 15.75^b^±0.04 | 14.95^cde^±0.13 | | 6.96^cde^±0.08 | | | 6.53^d-h^±0.08 | | | | | 0.94^k-m^±0.05 | | | | | 2.8^c^±0.08 | | | | | 11.37^d^±0.01 | | | | | 18.0 9^d^±0.01 | | | | |
| *A.altissima*.site2 | 14.74^efg^±0.08 | 14.67^ef^±0.17 | | 6.81^d-g^±0.24 | | 6.42^e-h^±0.13 | | | | 1.41^h^±0.11 | | | | | 2.4^e^±0.05 | | | | | 11.3^d^±0.005 | | | | | 15.08^d^± 0.01 | | | | |  |  |
| *A.altissima*.site3 | 15.42^bc^±0.06 | 14.55^b^±0.09 | | 7.86^b^±0.24 | | 6.50^d-h^±0.21 | | | | 1.60^g^±0.02 | | | | | 3.6^a^±0.01 | | | | | 13.53^d^± 0.01 | | | | | 18.7^d^± 0.01 | | | | |  |  |
| *A.altissima*.site4 | 21.51^a^±0.78 | 14.25^bcd^±0.10 | | 8.89^a^±0.33 | | 6.7^d-h^±0.12 | | | | 1.50^gh^±0.11 | | | | | 3.2^b^±0.07 | | | | | 17.38^d^±0.009 | | | | | 21.69^d^±0.009 | | | | |  |  |
| *S.babylonica*.site1 | 14.52^ef^±0.03 | 14.54^ef^±0.01 | | 6.48^d-h^±0.08 | | 6.36^f-h^±0.09 | | | | 0.81^no^±0.03 | | | | | 3.05^c^±0.1 | | | | | 12.86^d^±0.008 | | | | | 12.67^d^±0.01 | | | | |  |  |
| *S.babylonica*.site2 | 14.38^ef^±0.03 | 14.51^ef^±0.03 | | 6.39^e-h^±0.09 | | 6.28^gh^±0.04 | | | | 0.6^pq^±0.07 | | | | | 0.8^m-o^±0.06 | | | | | 12.17^d^± 011 | | | | | 10.98^d^±0.007 | | | | |  |  |
| *S.babylonica*.site3 | 14.41^ef^±0.02 | 14.55^ef^±0.03 | | 7.02^cd^±0.20 | | 6.21^h^±0.07 | | | | | 1.1^i-k^±0.07 | | | | | 3.3^b^±0.06 | | | | | 18.09^d^± 0.01 | | | | | 13.54^d^±0.009 | | | | |  |
| *S.babylonica*.site4 | 14.59^ef^±0.05 | 14.74^ef^±0.04 | | 6.9^c-f^±0.22 | | 6.48^e-f^±0.07 | | | | | 1.1^ij^±0.08 | | | | | 3.4^b^±0.01 | | | | | 19.07^d^± 0.01 | | | | | 13.94^d^±0.016 | | | | |  |
| *M.alba*.site1 | 14.2^f^±0.03 | 14.34^f^±0.02 | | 6.7^d-h^±0.22 | | 6.31^f-h^±0.07 | | | | | 0.6^op^±0.05 | | | | | 1.03^i-m^±0.02 | | | | | 20.5^d^±0.008 | | | | | 120.42^b^±0.005 | | | | |  |
| *M.alba*.site2 | 14.16^f^±0.06 | 14.28^f^±0.02 | | 6.52^d-h^±0.07 | | 6.18^h^±0.12 | | | | | 1.5^gh^±0.18 | | | | | 1.9^f^±0.06 | | | | | 15.98^d^±0.004 | | | | | 75.02^c^± 0.01 | | | | |  |
| *M.alba*.site3 | 14.24^f^±0.05 | 14.31^f^±0.05 | | 6.85^c-g^±0.11 | | 6.57^d-h^±0.37 | | | | | 2.2^e^±0.03 | | | | | 2.5^d^±0.07 | | | | | 24.28^d^± 0.01 | | | | | 149.93^a^±0.02 | | | | |  |
| *M.alba*.site4 | 14.43^ef^±0.02 | 14.46^ef^±0.05 | | 7.39^bc^±0.19 | | 6.31^fgh^±0.03 | | | | | 2.3^e^±0.04 | | | | | 2.3^e^±0.02 | | | | | 24.94^d^± 0.01 | | | | | 164.33^a^±0.10 | | | | |  |
|  |  |  | |  | |  | | | | |  | | | | |  | | | | |  | | | | |  | | | | |  |

| a) *M. alba* | b) *A .altissima* | c) *S. babylonica* |
| --- | --- | --- |
| **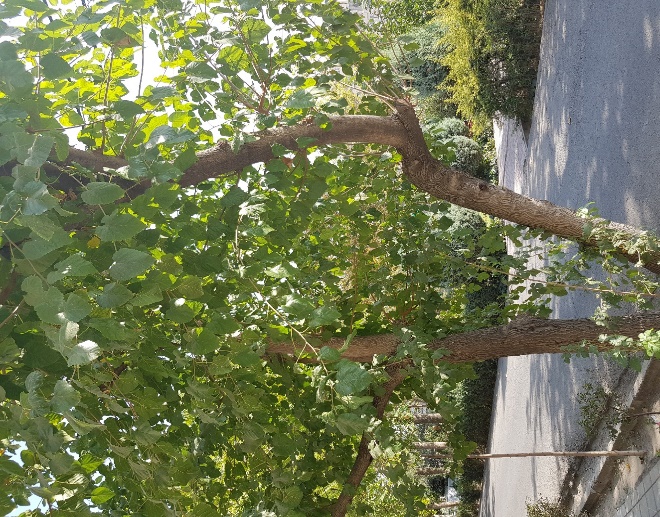** | 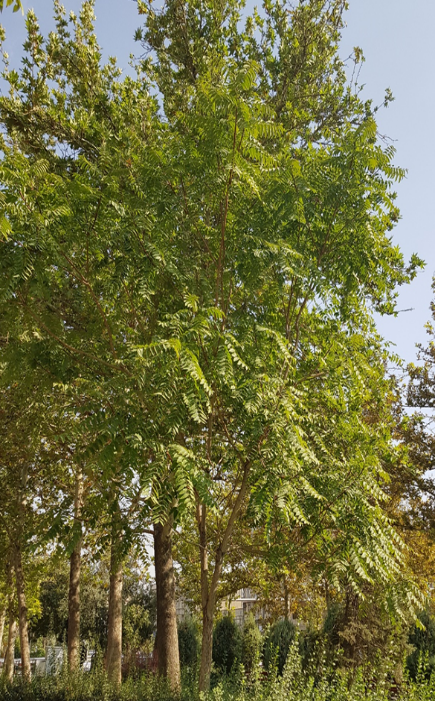 | **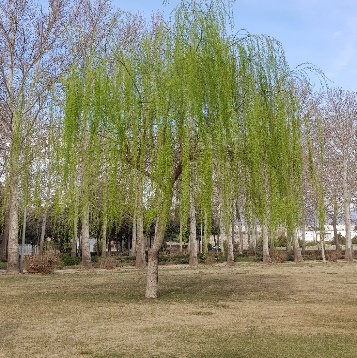** |

**Figure S1.** The trees of *M. alba* (a), *A. altissima* (b), and *S. babylonica* (c) at Sites 1 (Mirdamad Street), 2 (Mirza Babaei Street), 3 (Kargar Shomali Street), and 4 (Azadi Bus Station Street) of Tehran City (photographs were taken by Hamed Dadkhah-Aghdash).

| **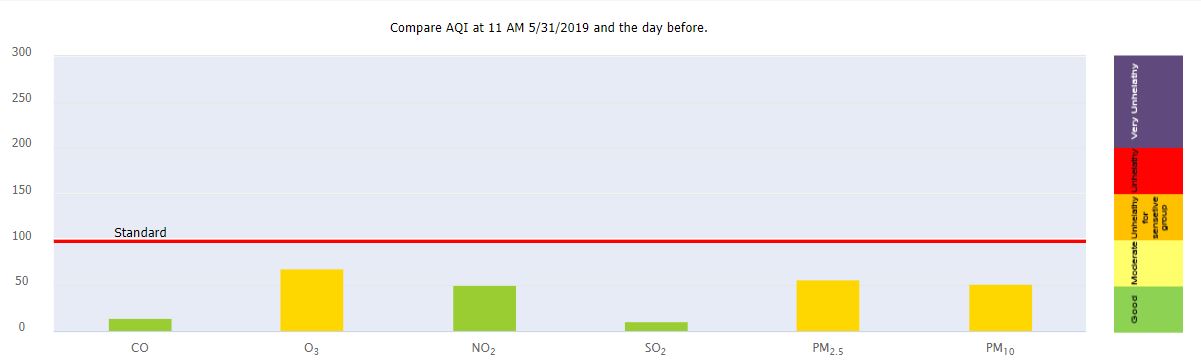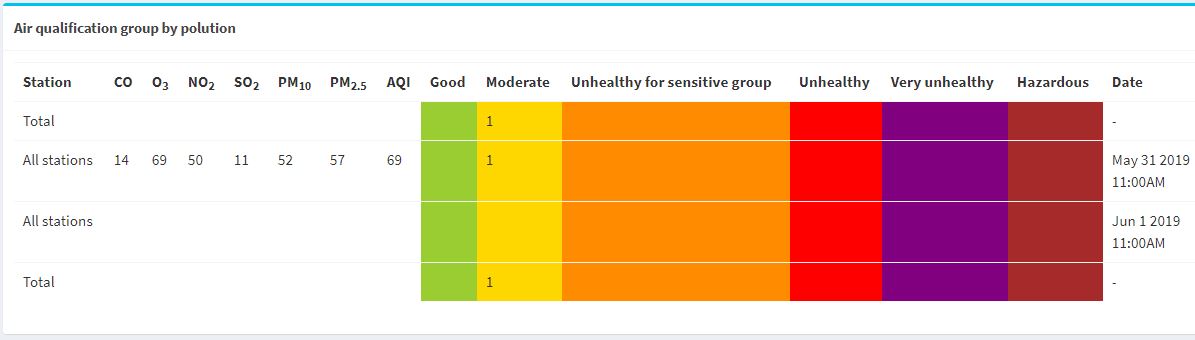**  **Figure S2.** Concentration of air pollutants in Tehran City at 11 AM 31 May 2019  (<http://www.airnow.tehran.ir>) |
| --- |
| **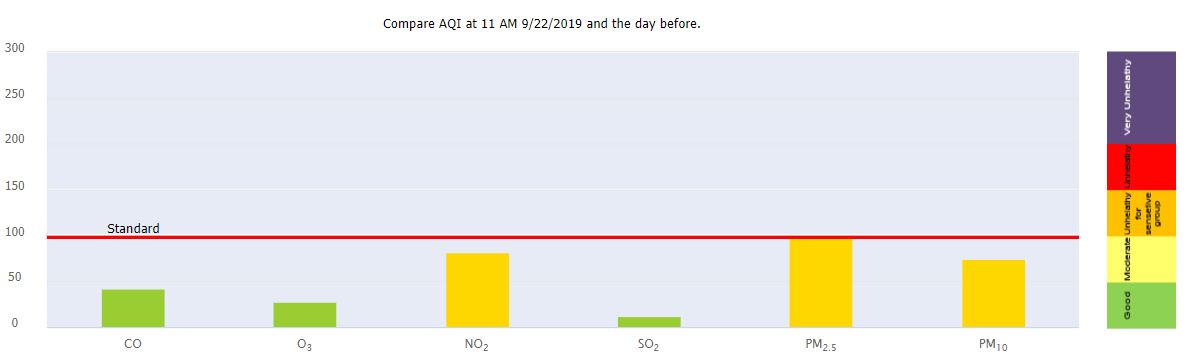** |
| **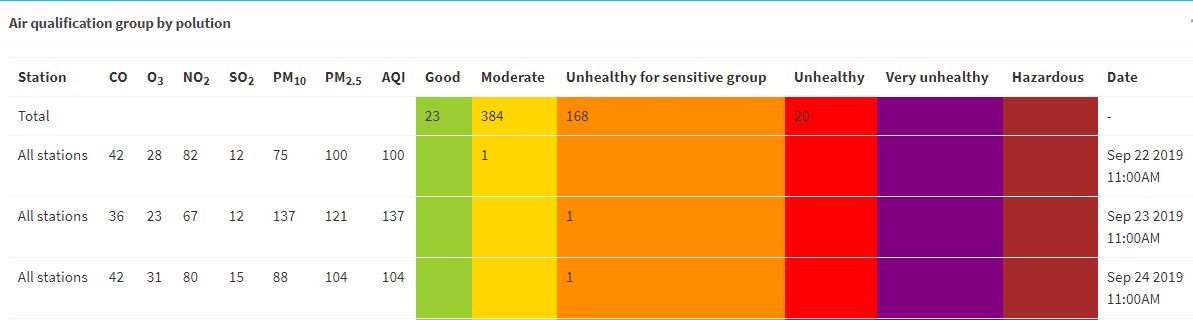** |
| **Figure S3.** Concentration of air pollutants in Tehran City at 11 AM 22 September 2019  )<http://www.airnow.tehran.ir>) |
| 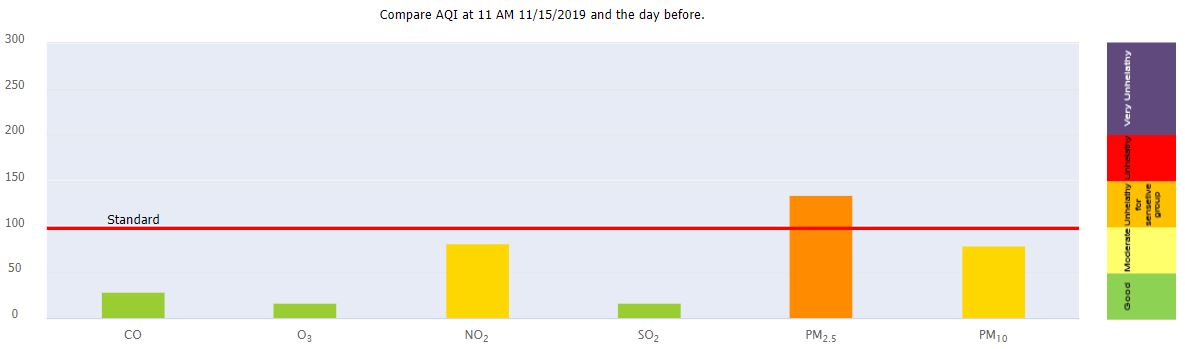 |
| 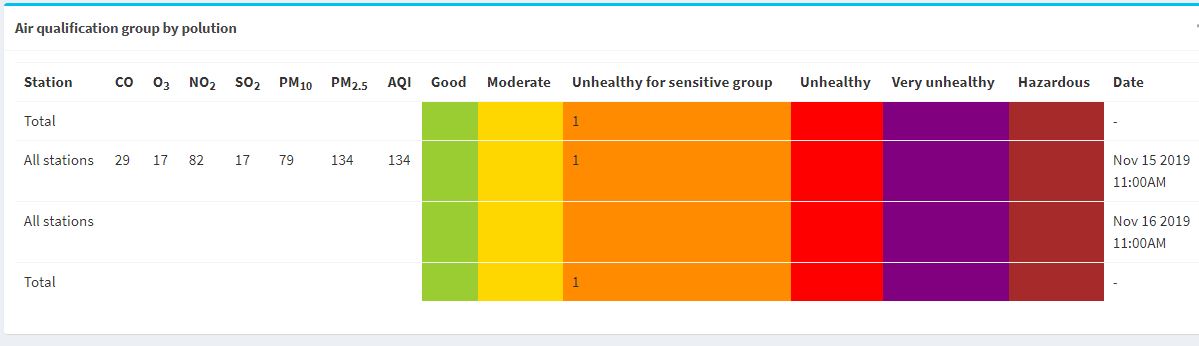 |

**Figure S4.** Concentration of air pollutants in Tehran City at 11 AM 15 November 2019

(<http://www.airnow.tehran.ir>)
